# Removal of Fatty Fibroma

**Published:** 1881-07

**Authors:** Mark L. Frey


### III.—REMOVAL OF FATTY FIBROMA, ANTISEPTICALLY.

REPORTED BY MARK L. FREY, D.V.S.

On the fifteenth of February a gray horse, sixteen hands high and weighing about sixteen hundred pounds, was brought to the hospital of the Columbia Veterinary College. The animal was quite lame, suffering from a fatty fibroma, involving the anterior spinatus, mastoid-humeralis and sterno-premaxillaris muscles, and interfering with deglutition. The fibroma had been increasing in size for the past nine months, and was probably caused by undue pressure of the collar. Blisters and setons had been applied, but without success.

The animal was secured and etherized, a long incision was made, and the tumor dissected out by Dr. Walton, the House Surgeon. It weighed four pounds. Carbolized spray was used; several arteries were ligated. The wound was brought together by quill sutures; and a compress, consisting of a moistened sponge, was placed over the part and held in position by a bandage passing over the shoulder.

Antiseptic treatment was kept up, stimulants used, and the animal made a splendid recovery, being discharged March 10th.

